# Physicochemical Composition and Antioxidant Activity of Five Gari Processed from Cassava Roots (*Manihot esculenta Crantz*) Harvested at Two Different Maturity Stages and Two Seasons

**DOI:** 10.1155/2023/4779424

**Published:** 2023-10-25

**Authors:** Alphonse Laya

**Affiliations:** ^1^Department of Biology Faculty of Science, University of Maroua, P.O. Box 814, Maroua, Cameroon; ^2^Fruit and Vegetable Technology Department, CSIR-Central Food Technology Research Institute, Mysuru 570020, India

## Abstract

*Gari* is a partially gelatinized roasted fermented granular white or yellowish product made from storage roots of cassava. It is consumed as fast foods in many countries across the world. Physicochemical composition, particle size, colour, and antioxidant activities of five *gari* (92/0326, 96/1414, IRAD4115, EN, and AD) processed from fresh storage roots harvested at 12 months after planting (MAP) and 15MAP compared to four (4) commercial *gari* (M1, M2, M3, and M4) were evaluated. The analytical results revealed that colour value *b*^∗^ and particle size varied significantly (*p* < 0.05) among the *gari* samples. Bound flavonoid contents were lower than free flavonoids (3.93 to 10.50 mgQE/100 g and 2.40 to 8.85 mgQE/100 g, respectively). Fourier transform infrared confirmed the functional groups in all *gari* samples. The antioxidant activity of the bound phenolics showed significantly (*p* < 0.05) higher DPPH scavenging ability than free phenolics (*gari* M2: 2.70 *μ*gTE/g). Similarly, the bound phenolics showed significant (*p* < 0.05) variation of HRSA scavenging activity (0.18-35.09 *μ*gTE/g). However, the best HRSA scavenging activity was found with bound phenolics of *gari* 96/1414, whereas HRSA scavenging activity was not detected in *gari* 92/0326, 96/1414, and AD. The value of ABTS scavenging activity of *gari* varied significantly (*p* < 0.05) from 20.60 to 30.17 *μ*gTE/g and from 20.70 to 34.39 for free and bound phenolics, respectively, while free phenolics showed higher FRAP value (7.97 mgTE/g) than the bound phenolics (4.59 mgTE/g). Additionally, phenolics and antioxidant activities showed significantly (*p* < 0.05) a positive correlation. The present study has provided an insight into the physicochemical composition, bioactive compounds, and antioxidant activities of various *gari* processed at different season and maturity period of harvesting. It reveals that consumers of cassava *gari* can get health benefits apart from the nutritional values.

## 1. Introduction

Cassava (*Manihot esculenta* Crantz), which belongs to the family Euphorbiaceae is cultivated through the tropics. It is known as a staple food in many parts of the world, mostly in the developing countries. Their storage roots provided carbohydrates for more than 2 billion people in the tropics [1]. It serves also as an industrial raw material in pharmaceutical and chemical industries. Cassava leaves are consumed as green vegetable in various forms as well as used for medicinal purposes [2, 3]. It is also a source for biofuel as well as animal feed. In Cameroon, cassava was among the staple food throughout the country with the exception of the Northern Region. The leaves of cassava are used to prepare various sauce or soup and *magani* [4]. In general, cassava storage roots may spoil quickly after harvest (two to three days), so it is recommended to process into products such *gari*, semolina, flour, fufu, tapioca, and starch. These products were traditional foods that are consumed widely across the country. Among these by-products, *gari*, a fermented product, appears to be mostly appreciated for consumption due to its nutritional quality and relative long shelf life comparable to other processed products of cassava storage roots [1, 5]. Processing cassava storage roots into *gari* usually involves several processing steps due to the need to remove the toxic, cyanoglucoside contents, inherent to the storage roots for its safe level. In addition, *gari*, a fermented granulated food, had prebiotic and probiotic beneficial activity in the human body [6–8]. In recent decades, natural dietary antioxidants had gained much attention due to their importance to protect body human against noncommunicable diseases such as obesity, diabetes, cancer, and hypertension [3, 9–11]. Among these compounds, phenolic is known to be the most prominent natural dietary antioxidants widely distributed in cassava plants. However, the quality of the phenolic compounds may be altered during processing of raw material to process some nutritional products by using grinding, heating, sieving, etc. Hence, their bioactivity decreased and affected directly the antioxidant activities due to the substantial losses of antioxidant components [12]. In addition, the distribution of particle size in food processed may be also a critical parameter for organoleptic quality of the products. However, fermentation by microorganisms could lead to the structural breakdown of cell walls, leading to the synthesis of new metabolites and liberation of various other bioactive ingredients with more bioactivity. The functional properties and nutritional quality of *gari* produced at different periods compared to commercial gari have been studied by Laya et al. [1]; however, an evaluation of antioxidant activity of different fractions of phenolics from this *gari* has been not carried out.

Thus, to our best knowledge, studies composed of total free and bound phenolic compounds in cassava *gari* were not reported. Nowadays, there was no information about the antioxidant activity of cassava *gari* related to their phenolic compounds, especially the free and bound phenolic contents. Thus, assessed polyphenolics and their antioxidant activities were important in order to know whether consumers of cassava *gari* could get health benefits to improve their well-being.

Therefore, the present study is aimed at assessing the physicochemical, bioactive compounds, and antioxidant properties of the processed cassava *gari* at different times of the harvest compared to local commercial *gari* collected in the local markets in order to know whether gari samples processed from different varieties at different maturity periods and seasons could be reliable as a source of nutritional value with a high organoleptic property, antioxidant compounds, and antioxidant activity for consumers.

## 2. Materials and Methods

### 2.1. Chemicals and Reagents

All standards (97% > purity) such as quercetin, tannic acid, and salicylic acid (98% > purity) for Trolox, ABTS, DPPH, and TPTZ were obtained from Sigma-Aldrich (Bengaluru, India). Milli-Q water (Millipore, USA) was used for all experiments. Other chemicals (99% > purity) and reagents (95-98% > purity) used were of analytical grade purchased from Sisco Research Laboratory (SRL) from Mumbai (India).

### 2.2. Plant Materials

Fresh matured storage roots of five cassava varieties named 92/032 and 96/1414 from IITA (International Institute of Tropical Agriculture, Nigeria) and 4115 from IRAD4115 (Institute of Agricultural Research for Development, Adamawa Region of Cameroon) were improved varieties, EN was local sweet variety, and AD was also local bitter variety obtained from experimental field in Tokombéré subdivision (10° 54′0.0^″^ north latitude and 14° 12′0.0^″^ east longitude), Maroua, Cameroon. Their storage roots were harvested in dry season at twelve months after planting (MAP) and in rainy season at fifteen MAP, respectively.

### 2.3. Processing of Cassava Storage Roots into Gari

The cassava storage roots were processed into *gari* using modified method described by Agbor-Egbe and Mbome [13] and Amamgbo et al. [14].

### 2.4. Colour Measurement

The colour of *gari* sample was determined using the *L*^∗^ *a*^∗^ *b*^∗^ colour notation system with spectrophotometer Konica Minolta (CM-5, Tokyo, Japan). Forty (40) grams of sample was weighed into a dish, and the colour was measured. The chromameter was calibrated with a standard white tile. The instrument was set at illuminant D65. Colour measurement was replicated three times.

### 2.5. Scanning Electron Microscopy (SEM)

Samples of gari were prepared as intact specimens to observe the structure surface. The gari samples were mounted onto a stub using conductive tape and sputter coated for 90 s with gold/palladium. All samples were viewed in a Leo Electron Microscopy (model Leo 435VP, 1995; Cambridge, UK) scanning electron microscope.

### 2.6. Particle Size

Fifty (50) grams of *gari* was sieved through a set of graded Tyler sieves of aperture sizes 700, 500, 300, 250, 150, 100, 60, 40, 20, and 10 *μ*m using a Microtrac (analysis mode: S3000/3500, Germany) set zero time 30 seconds and run time 10 seconds for the total number of runs 3. Fractions retained on each sieve were then weighed and used to calculate the particle size distribution percent of the *gari* samples.

### 2.7. FTIR Spectroscopy Analysis

Fourier transform infrared (FTIR) spectrophotometer was used to analyze the functional groups of the active components present in methanolic extract based on the peak values in the region of IR radiation. Methanolic extracts (10 *μ*L) of *gari* sample were characterized using Fourier transform infrared spectrometer Tensor II with platinum ATR (Tensor II, FTIR-Bruker, Ettlingen, Germany) for FTIR spectrum measurement, 16 scans with the resolution of 4 in the frequency range of 400 to 4000 cm^−1^. The instrumental control was carried out using OPUS software (OPUS v. 7.0 for Microsoft, Bruker Optics, Ettlingen, Germany).

### 2.8. Polyphenolic Extraction

Free and bound phenolic compounds were extracted following the method of Chen et al. [15] with some changes. Shortly, 20 mL of methanol was added into the fine ground *gari* powder (300 mg) in triplicate for each analysis and mixed overnight by placing over dancing shaker at room temperature (25°C). The mixture was centrifuged at 10,000 rpm for 20 min at 4°C. The extraction solution was filtrated with Whatman no. 1. Then, the residue was washed two times with 5 mL of methanol HPLC grade and then centrifuged. The supernatants were combined and centrifuged at the same conditions described above and filtrated with Whatman no. 1. The solvent was evaporated at 45°C to dryness and dissolved in methanol (HPLC grade) before keeping at -20°C until analysis. For bound phenolic content, the residue resulting from free phenolic extraction was mixed with 10 mL NaOH (2 N) solution and boiled in shaking water bath at 50 rpm for 30 min before adding 5 mL HCl (2 M), and it was incubated for 60 min at 60°C. The extraction mixture was cooled, and 10 mL of ethyl acetate was added into the solution. After 1 h shaking, the solution was filtered and the ethyl acetate fraction was centrifuged as described above. The residue obtained after filtration was mixed more time with 10 mL of ethyl acetate and centrifuged at the same conditions as described above before filtrating through Whatman no. 1. The combined supernatant was evaporated at 45°C to dryness using rota vapor. The residue obtained was dissolved in methanol HPLC grade and centrifuged as described previously. The methanol extract was kept at -20°C until analysis.

### 2.9. Flavonoid Content

Free and bound flavonoid contents were determined as described by Xiong et al. [16] with some modifications using multimode reader (Tecan SPARK 10M, V1.2.20, Austria). In brief, 20 *μ*L of sample was mixed with 20 *μ*L of 5% sodium nitrite and of 2% aluminum chloride. Then, the mixture was incubated for 3 min before adding 50 *μ*L NaOH (0.5 M). The solution mixture was mixed again for 40 s and incubated for 6 min. Then, 30 *μ*L of 2% sodium acetate was added in each well and incubated for 40 min in the dark place. The absorbance was recorded at 510 nm using multimode reader (Tecan SPARK 10M, V1.2.20, Austria). The quercetin was used as a standard.

### 2.10. Total Tannin Content

Total tannin content was determined using the vanillin reagent according to Gaytan-Martínez et al. [17] with some modifications using multimode reader. First, 20 *μ*L of the sample was mixed with 120 *μ*L of vanillin reagent (4% vanillin in methanol). The mixture was incubated for 3 min, and after that, 30 *μ*L of concentrated HCl (36.5%) was added. The microplate was covered with aluminum foil and incubated for 20 min in the dark. The absorbance was taken at 500 nm in multimode reader (Tecan SPARK 10M, V1.2.20). The tannic acid was used as a standard.

### 2.11. Antioxidant Activity Assay

#### 2.11.1. Ferric Reducing Antioxidant Power (FRAP)

The FRAP was carried out according to the method described by Feregrino-Pérez et al. [18] with minor changes. Using a micropipette, 20 *μ*L of the sample or blank was added to 120 *μ*L of the FRAP reagent in a 96-well microplate and the mixture was shaken for 50 s and then incubated for 40 min. The absorbance was recorded at 595 nm with multimode reader (Tecan SPARK 10M, V1.2.20), and the results were determined using the Trolox as a standard.

#### 2.11.2. 2,2-Diphenyl-1-Picrylhydrazyl (DPPH)

The DPPH radical scavenging activity of the cassava gari was carried out according to the method described by Feregrino-Pérez et al. [18] with some modifications. To the sample solution (40 *μ*L), 150 *μ*L of DPPH (1 mM) was added in a 96-well microplate (New York, USA). The solution was mixed for 40 s using multimode reader (Tecan SPARK 10M, V1.2.20, Austria) and incubated for 40 min in the dark place. After incubation, the absorbance was recorded at 517 nm with multimode reader (Tecan SPARK 10M, V1.2.20). The results were evaluated from the Trolox as a standard.

#### 2.11.3. 2,2-Azinobis-(3-Ethylbenzothiazoline-6-Sulfonic Acid) (ABTS)

ABTS radical scavenging was performed as previously reported by Wu et al. [19] with some modifications using multimode microplate reader (Tecan SPARK 10M, V1.2.20, Austria). The reaction started by adding 20 *μ*L of the extract or blank into 150 *μ*L of ABTS^+^ solution. The microplate was shaken for 50 s before incubation at room temperature for 40 min in the dark. The absorbance was taken at 734 nm, and the results were evaluated from the Trolox as a standard.

#### 2.11.4. Hydroxyl Radical Scavenging Activity (HRSA)

HRSA was evaluated as previously described by Feregrino-Perez et al. [20] with some modifications. Shortly, 50 *μ*L of FeSO_4_ (9 mM), 50 *μ*L of H_2_O_2_ (0.03%, *v*/*v*), 50 *μ*L of salicylic acid-ethanol solution (9 mM), and 50 *μ*L of extract were mixed in 96-well microplate (New York, USA) and incubated for 40 min in the dark place. The absorbance was taken at 510 nm with multimode microplate reader. Trolox was used as a control. HRSA was calculated from the Trolox as a standard curve plotted with seven concentrations (5-1000 *μ*g/mL).

### 2.12. Statistical Analysis

ANOVA was carried out using Statgraphics software (version 16). Tukey's (HSD) test was used to determine any significant difference between different gari, and significance was accepted at level *p* < 0.05. Correlation and principal component analysis (PCA) were done to establish the relationship between antioxidant activities and phenolic compounds. The results were expressed as means ± standard deviation. The experiments were carried out in four replications.

## 3. Results and Discussion

### 3.1. Physical Properties of Gari Samples

#### 3.1.1. Colour

Colour is one important factor attribute that determines the quality of *gari*. It is also one of the key quality attribute that influence consumer's behavior, choices, and perceptions [21]. The *L*^∗^ *a*^∗^ *b*^∗^ colour notation was used to describe the colour of the *gari* samples, where *a*^∗^ means the degree of redness or greenness and *L*^∗^ and *b*^∗^ indicate the degree of lightness and the intensity of yellowness, respectively [21]. The difference in the colour of the *gari* samples was very significant (*p* < 0.05) (Table 1). This difference observed in the colour of the *gari* samples obviously resulted from the fact that storage roots used for garification were harvested at different stages and seasons that affected the physiological changes. The *gari* produced from roots harvested at 12MAP and 15MAP had significantly affected the colour of gari, which was also greatly different from the commercial *gari* samples (Table 1). Thus, period of the harvest seems to have significantly (*p* < 0.05) affected the colour of the *gari* samples. The values *b*^∗^ of *gari* produced at 12MAP varied from 29.99 to 37.87 while those at 15MAP varied from 25.93 to 29.06 and are significantly less than that of commercial *gari* (26.17-48.56). The value *b*^∗^ obtained in the present study was slightly similar as observed by Oduro et al. [22] in their work, however, higher than that reported by Chuzel et al. [23] who recommended the range of 17-21 only on small-scale *gari* samples. The highest *b*^∗^ value of commercial *gari* sample M1 may be due to addition of colour additives as reported by Oduro et al. [22]. Generally, producers of commercial *gari* used huge amount additives during garification.

#### 3.1.2. Scanning Electron Microscopy (SEM)

**Figure 1:**
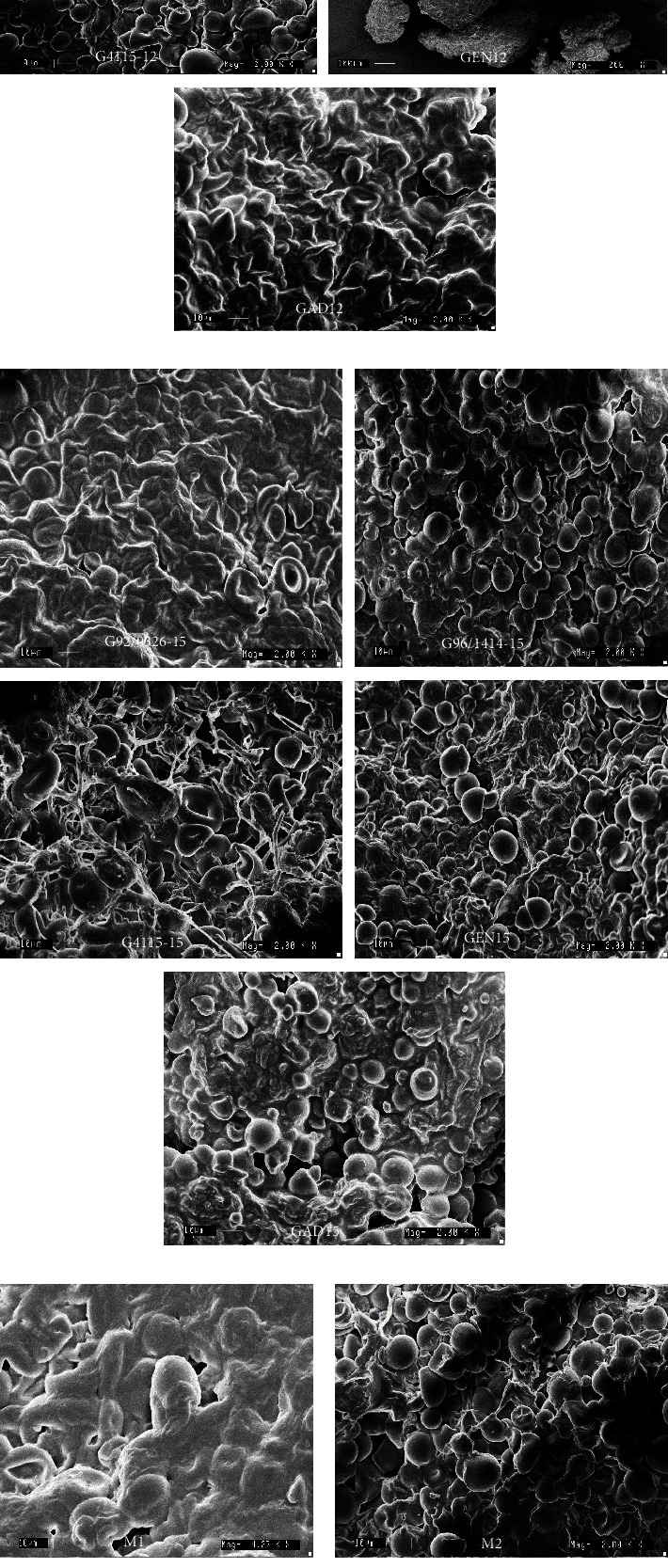
SEM of different gari samples: (a) produced gari at 12MAP (dry season); (b) processed gari at 15MAP (rainy season); (c) commercial gari.

Starch granule damage morphology of *gari* samples was studied using a SEM. Starch granule damage morphology from *gari* produced at 12MAP had a different morphology (Figure 1(a)) compared to those gari produced at 15MAP (Figure 1(b)) as well as commercial *gari* (Figure 1(c)). The starch granules of *gari* samples had broken with fissures, microholes, and rough surfaces, while some granules were clumped together and showed a cluster with large aggregates (Figure 1(b)). This result suggests that fiber matrix structure was damaged during fermentation by microorganism activity as reported by Hidayat et al. [24], hence starch breakdown in small size granules. On the other hand, the modified starch may have high amylose content; thus, excessive leaching of this amylose causes the starch granules to collapse resulting in gelatinization [25]. This indicates more severe damage of starch granules during fermentation and roasting process. The modification of starch morphology may improve the functionality of gari including water absorption, solubility, and swelling power. However, the clumped granules may reduce the water absorption capacity and solubility of the *gari* samples. It was reported that the changes of structure and morphology of starch granules impacted deeply on the pasting properties and thus increased in starch digestibility due to damaged granules [26]. Furthermore, the smaller granules of *gari* may expand to a great surface area and better digestion due to contact of food with enzyme activity [27]. It is obvious that the damage of starch granules depends on the type of *gari* as affected by varietal differences (Figure 1).

#### 3.1.3. Particle Size Distribution

Apart from the appearance as among the factors which affect the quality of *gari*, particle size seems to be one of the most important for consumers [28]. On the other hand, in nutrition, particle size has significant effect on food digestibility in the gastrointestinal system [29]. Thus, study distribution of particle size of ready food for consumer is a challenge for the food industry in order to offer a high organoleptic quality. In this study, the results of particle size of *gari* samples are shown in Table 2. On mesh sizes 10 *μ*m and 20 *μ*m, the percentage retention varies from 100 to 99.39 and 100 to 90.97%, respectively. Mesh sizes 40 and 60 *μ*m have percentage retention varying from 100 to 79.9.66% and 100 to 63.41%, respectively. However, on mesh sizes 100 and 150 *μ*m, the percentage retention varies from 100 to 52.62 and 100 to 52.33%, respectively. On mesh sizes 250 and 300 *μ*m, the percentage retention varies from 99.60 to 42.69 and 97.36 to 30.76%, respectively. While on mesh sizes 500 and 700 *μ*m, the percentage retention varies from 84.35 to 36.25 and 77.51 to 5.75%, respectively (Table 2). Variation in particle size distribution of gari sample size may be due to the differences in the grating procedure, roasting process, and the period of fermentation of the cassava mash compared to commercial *gari*. In fact, fermentation has been shown to affect particle size [22]. The variation in particle size may also be attributed to the stage of harvest cassava storage roots to process into *gari* and variety as well. The appearance is one factor that dictates the quality of *gari*; however, the particle size distribution is the most important for consumers because they will most likely have high preference for fine particles [30]. Furthermore, the separation of particles on the basis of particle size distribution may help to provide some information on functional properties of products processed including water hydration and textural property [29].

### 3.2. FTIR Spectroscopy

**Figure 2:**
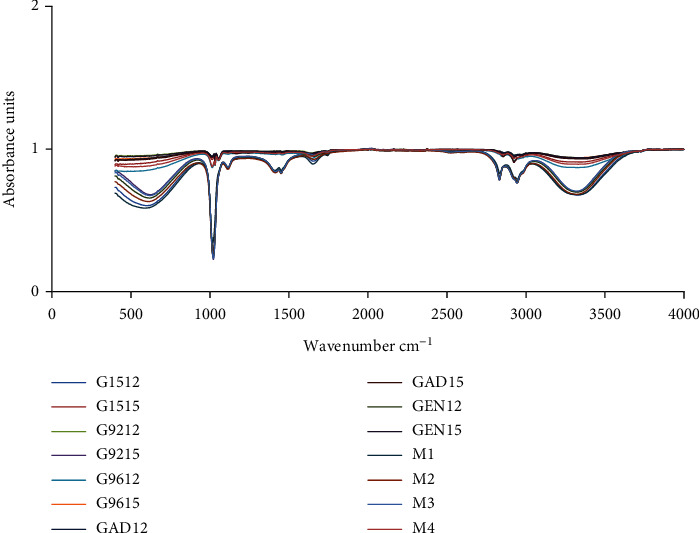
FTIR spectra of methanolic extracts of all gari samples. G1512: gari 4115 produced at 12MAP (months after plantation, dry season); G1515: gari 4115 produced at 15MAP (raining season); G9212: gari 92/0326 produced at 12MAP; G9215: gari 92/0326 produced at 15MAP; G9612: gari 96/1414 produced at 12MAP; G9615: gari 96/1414 produced at 15MAP; GAD12: gari AD produced at 12MAP; GAD15: gari AD produced at 15MAP; GEN12: gari EN produced at 12MAP; GEN15: gari EN produced at 15MAP; M1, M2, M3, and M4: commercial gari.

Fourier transform infrared (FTIR) is the most powerful tool for identifying the types of functional groups (chemical bonds) present in *gari*. The FTIR spectra related to studied samples are shown in Figure 2. The bands identified in samples displayed in Table 3 revealed the presence of many functional groups in methanolic extracts of gari. The FTIR spectrum confirmed the presence of phenols, amino acids, amides, carboxylic acids, alkanes, aliphatic esters, secondary alcohols, sulfur compounds, and mono substituted alkenes in methanolic extract of *gari* samples. The functionality of *gari* including water absorption and solubility may be influenced by proteins, amino acids, and other functional groups of the gari revealed by FTIR (Table 3). On the basis of FTIR spectra, there was partial gelatinization that occurred disrupting the ordered crystalline structure of amylopectin of starch [25]. Finally, possible leaching of amylose resulting from heat treatment increases the chances of the different molecules interacting with each other including hydrogen bonding and amylose [25], and this interaction may improve the swelling power of gari which is the one parameter influencing the gari consumption by consumers.

### 3.3. Polyphenolic Compounds of Methanolic Extracts of Gari Samples

#### 3.3.1. Flavonoid Contents

As shown in Table 4, there was significant (*p* < 0.05) variation of free and bound flavonoid content in *gari* samples. However, the highest value (10.50 mgQE/100 g) of free flavonoids was shown by commercial gari M1, while the lowest value (3.93 mgQE/100 g) was observed in *gari* AD produced at 15MAP (Table 4). These results suggest that a variety may affect the flavonoid levels in *gari* samples. The highest value of free flavonoids could be attributed to variety and season of the harvest periods of storage roots used for garification [31]. Variation of flavonoid contents in *gari* samples could be attributed to processing methods as shown by commercial gari. The lowest value (2.42 mgQE/100 g) of bound flavonoid was shown by *gari* EN produced at 12MAP while the highest value was obtained with gari 96/144 produced at 15MAP (Table 4). However, values of flavonoid contents in all *gari* samples were lower compared to the values obtained (3.17-5.19 g/100 g) from two types of *gari* consumed in Nigeria [32].

#### 3.3.2. Total Tannin Contents (TTC)

The TTC varied significantly (*p* < 0.05) in gari samples (Table 4). For free tannin content, the highest value (4.59 mgTAE/100 g) was shown by *gari* EN produced at 12MAP whereas the commercial gari M1 showed the lowest concentration (0.4 mgTAE/100 g). The *gari* produced at 15MAP recorded the highest value of tannin than those produced at 12MAP that suggest the effect of harvesting stages. Both bound and free TTC are significantly higher in *gari* produced at 12MAP and 15MAP compared to commercial gari, suggesting variety and processing methods had significant (*p* < 0.05) effect. The tannin contents observed in this study were higher than those (0.02-0.05 mg/100 g) obtained by Oluba et al. [33]. However, they did not mention in their study whether the phenolics of the samples were free or bound fraction. The values of tannins (0.18-0.19 g/100 g) observed by Rocchetti et al. [34] were higher than the values obtained in the present study. In addition, Airaodion et al. [31] reported higher values (0.22-0.30 g/100 g) of tannins in two types of *gari*.

### 3.4. Antioxidant Activities

#### 3.4.1. Ferric Reducing Antioxidant Power (FRAP)

FRAP of free and bound extract *gari* samples is shown in Table 5 with values ranging from 2.78 to 7.97 mgTE/gdw and 0.78 to 4.59 mgTE/gdw, respectively. For free extracts, EN *gari* produced at 12MAP had significantly (*p* < 0.05) higher FRAP compared to the other *gari* samples, while AD gari extracts had the lowest FRAP value (2.78 mgTE/gdw). The 92/0326 *gari* extracts of bound fraction showed the highest FRAP value, while AD *gari* had the lowest FRAP value. These variations of FRAP values of free and bound fractions of phenolics were reported by Rocchetti et al. [34] on cooked gluten-free pasta. However, FRAP activity was significantly higher in free fraction phenolics compared to the bound fraction. Similar result was obtained by Laya and Koubala [2], who found that the free form of phenols in cassava leaves showed the highest FRAP activity than bound form of polyphenolics. The results suggest that free phenolics may have the ability to reduce the potassium ferricyanide (Fe^3+^) into potassium ferrocyanide (Fe^2+^) than the bound fraction of phenolics. On the other hand, dry heating may improve the phenolic bioactivity, which helps to enhance the antioxidant activity [35].

#### 3.4.2. Ferric Reducing Antioxidant Power (DPPH)

DPPH radical scavenging capacity of free and bound extracts of *gari* samples is shown in Table 5 with values ranging from 3.76 to 13.05 *μ*gTE/gdw and 2.70 to 22.01 *μ*gTE/gdw, respectively. Free fraction of phenolics of AD gari produced at 12MAP showed significantly (*p* < 0.05) the highest value of DPPH radical scavenging activity compared to the other *gari* samples tested while the EN gari extracts had the lowest value of DPPH radical scavenging capacity (3.76 *μ*gTE/gdw). The bound fraction of phenolic extracts of commercial *gari*, M4, showed significantly (*p* < 0.05) the highest value of DPPH radical scavenging capacity (22.01 *μ*gTE/gdw) followed by EN *gari* (19.63 *μ*gTE/gdw) produced at 15MAP, indicating the lowest antioxidant activity. Similar results were obtained on sweet corn in China and various breads by Zhang et al. [36] and Verardo et al. [37], respectively.

#### 3.4.3. 2,2-Azinobis-(3-Ethylbenzothiazoline-6-Sulfonic Acid) (ABTS)

The ABTS activity of free and bound phenolic extracts of *gari* samples is shown in Table 5 with values ranging from 20.60 to 30.17 *μ*gTE/gdw and 20.70 to 34.39 *μ*gTE/gdw, respectively. The free fraction of phenolic extracts of AD gari produced at 15MAP showed significantly (*p* < 0.05) higher value of ABTS activity compared to the other *gari* samples, while the commercial *gari* extracts M4 had the lowest value of ABTS capacity (20.60 *μ*gTE/gdw), indicating the highest antioxidant activity. These results were in agreement with Airaodion et al. [31], who found that the bound phenolic compounds of various bread had higher antioxidant activity than free fraction of phenolics. The bound fraction extracts of phenolics of AD gari produced at 15MAP showed significantly (*p* < 0.05) the highest value of ABTS capacity (34.39 *μ*gTE/gdw) among the other *gari* samples while commercial gari M2 had a value of 20.70 *μ*gTE/gdw. The present results were similar to the antioxidant activity of bound and free fractions of phenolic compounds in sorghum obtained by Wu et al. [19]. The results may be due to the liberation of new metabolites and various antioxidant ingredients during the breakdown of plant cell walls through fermentation by microorganisms, which helps to increase the antioxidant activity [38].

#### 3.4.4. Hydroxyl Radical Scavenging Activity (HRSA)

The HRSA activity of free and bound extracts of phenolics of *gari* samples ranges from 2.58 to 14.80 *μ*gTE/gdw and 0.18 to 35.09 *μ*gTE/gdw, respectively. However, the HRSA activity of free fraction of phenolics (*gari* from 92/0326 and 96/1414 at 6MAP and AD at 9MAP) was not detected (Table 2). These results may suggest that variety differences and harvesting time of storage roots used to produce *gari* affect the quality of phenolic compounds. The free fraction of phenolics of commercial *gari* M4 showed significantly (*p* < 0.05) higher HRSA activity compared to the other gari samples, while the 4115 *gari* extracts produced at 12MAP had the lowest value of HRSA scavenging capacity (2.58 *μ*gTE/gdw). The bound fraction extracts of 96/1414 *gari* produced at 15MAP showed significantly (*p* < 0.05) the highest value of HRSA value (35.09 *μ*gTE/gdw) compared to the other *gari* samples, while commercial *gari* M3 had the lowest value (0.18 *μ*gTE/gdw), indicating the highest antioxidant activity. This result was in agreement with Laya and Koubala [2], who found that the bound fraction of phenolics in cassava leaves had higher antioxidant activity than free fraction of phenolics, and this activity varied significantly (*p* < 0.05) among *gari*. Similarly, Liyana-Pathiraka and Shahidi [39] reported that bound form of phenolic contents had higher antioxidant activity than free phenolic compounds in wheat. It was also reported that fermentation is effective at increasing phenolics and antioxidant activity [40–42].

Furthermore, there are many other parameters which imply in antioxidant activity; for example, the heated or processed products may increase the activity including the Maillard reaction or caramelization reactions which occur during heating involve reducing sugars reacting with amino acids, peptides, or proteins producing products that have potential antioxidant properties (including FRAP, DPPH, and ABTS) which may improve the gastrointestinal health [43–45]. These products act by many mechanisms, including metal chelation and free radical scavenging [44]. The Maillard reaction products are capable of binding metal ions and scavenging free radicals and can thereby improve the oxidative stability of lipids [46]. Additionally, many researchers reported also the effects of particle size on the bioactive phenolics and antioxidant activity. For example, Prasedya et al. [47] reported significantly stronger antioxidant activity including FRAP, ABTS, and DPPH in macroalgae samples with smaller particle sizes than coarser particle distribution. Similarly, Dziki et al. [48] found that the antioxidant activity of oat husk (by-product from oat processing) increased as the particle size of the micronized husk decreased. Overall, *gari* samples EN and M1 with smaller particle size distribution than others showed low to moderate antioxidant activity with some discrepancies which may be due to varietal effects. The results may be also different because bioactive phenolics were extracted in different fractions and the antioxidant activity was assessed in the form of free and bound which may affect deeply the present results. The results suggested that the matrix of sample and types of samples affect the bioactive compounds and consequently their antioxidant activities. The results are in agreement with Nabil et al. [49] who found that bioactive compounds and antioxidant activity (FRAP, DPPH, and ABTS) of cladode powder were differentially distributed according to particle size with some fractions having high antioxidant activity.

### 3.5. Correlation between Antioxidant Activity, Flavonoid, and Tannin Content

Table 6 shows the results of different correlations between antioxidant activity of bound and free phenolics and polyphenolic compounds (bound and free). High positive correlation (*r* = 0.638, *p* < 0.05) was found between FRAPf and FRAPb assays. Similarly, there was a significantly positive relationship (*p* < 0.05) between HSRAf and FRAPf (*r* = 0.595) and between DPPHf and FRAPb (*r* = 0.536) assays. In addition, a significant positive correlation was found between ABTSb and HRSAb (*p* < 0.05) as can be seen in Table 6. The results were in agreement with Zhang et al. [50], who found a significant relationship between ABTS, DPPH, and phenolics both bound and free forms of phenolic contents. However, there was no significant correlation between DPPH, FRAP, and flavonoid contents both free and bound forms of polyphenols. These results were in contrast with those obtained by Liyana-Pathiraka and Shahidi [39]. Free phenolic compounds showed a positive relationship (*p* < 0.05) between Tanf and HSRAb (*r* = 0.538) and ABTSb (*r* = 0.565). However, Flab showed only a significant positive correlation with Tanf (*r* = 0.644). The positive correlation of polyphenolics and their antioxidant activity was reported by Savlak et al. [51].

### 3.6. Principal Component Analysis (PCA)

**Figure 3:**
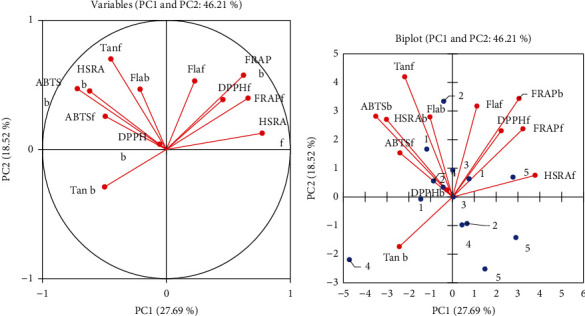
Principal component analysis (PCA) shows the relationship free, bound phenolics, and their antioxidant activities. Correlation (a) between variables and factors and biplot (b).

PCA showed clearly the separation between bound and free phenolic content and their antioxidant activities (Figure 3).  Figure 3(a) shows that the phenolic compounds and antioxidant activities quantified in the gari samples were reduced into two main components (PC1 and PC2). Free phenolics showed the opposite side of bound phenolic antioxidant activities except FRAPb (Figure 3(b)). F1 and F2 explain 46.21% of total data variance, with PC1 alone accounting for 27.69% of the observed variations. The variables which mainly contributed positively (F loading > 0.62) (Table 7) to PC1 are bound polyphenols forming antioxidants conferred by FRAP. However, PC2 accounts for 18.52% of the observed variations for free flavonoids; ABTS and DPPH contribute positively (F loading > 0.77) (Table 7). Thus, gari which has contributed mostly to the antioxidant capacities with their phenolics (bound and free) is divided into two groups according to F1 and F2 components (Figure 3(b)).

## 4. Conclusions

The present study revealed for the first time new knowledge on *gari* samples of bound and free phenolic compounds and their antioxidant activities. This study specifically demonstrated that *gari* samples were rich in phenolic contents and both free and bound forms showed antioxidant activity. However, the bound forms of phenolic compounds had a higher antioxidant activities than the free form. Thus, consumers of cassava gari can get health benefits to improve their well-being. It is recommended to harvest and process *gari* in dry season than in rainy season in order to maximize the antioxidant quality.

## Figures and Tables

**Table 1:** Colour of processed gari at 12MAP and at 15MAP compared to four (04) commercial gari collected in the local markets, Abattoir Maroua (Far North Region) and Bertoua (East Region) (Cameroon).

Harvest times	Gari	*L* ^∗^	*a* ^∗^	*b* ^∗^
12MAP	TMS92	69.06 ± 0.28^d^	8.77 ± 0.24^d^	37.87 ± 0.56^c^
15MAP		69.88 ± 0.40^cd^	6.62 ± 0.40^h^	28.90 ± 0.76^g^
12MAP	TMS96	69.90 ± 0.93^d^	7.43 ± 0.53^f^	29.99 ± 0.57^fg^
15MAP		73.94 ± 0.30^b^	5.01 ± 0.04^j^	25.93 ± 0.15^h^
12MAP	IRAD	69.58 ± 0.65^d^	7.89 ± 0.20^e^	33.18 ± 0.79^e^
15MAP		68.74 ± 0.42^de^	7.54 ± 0.19^f^	31.17 ± 0.23^ef^
12MAP	EN	78.29 ± 0.57^a^	3.67 ± 0.16^l^	25.39 ± 0.41^hi^
15MAP		74.01 ± 0.07^b^	5.45 ± 0.13^i^	25.49 ± 0.35^i^
12MAP	AD	67.70 ± 0.34^f^	8.74 ± 0.21^d^	31.64 ± 0.09^f^
15MAP		58.08 ± 0.07^g^	9.14 ± 0.43^c^	29.06 ± 0.45^g^
/	M1	68.39 ± 0.13^e^	11.12 ± 0.15^b^	48.56 ± 0.69^a^
/	M2	70.57 ± 0.21^c^	6.79 ± 0.21^g^	36.44 ± 0.73^d^
/	M3	67.05 ± 0.82^ef^	11.74 ± 0.21^a^	48.35 ± 0.52^b^
/	M4	70.46 ± 0.24^c^	4.20 ± 0.27^k^	26.17 ± 0.68^h^

12MAP: 12 months after planting (dry season); 15MAP: 15 months after planting (raining season); /: unknown months of the harvests. M1, M2, M3, and M4 were commercial gari collected from *Abattoir Maroua* and *Bertoua* markets, respectively. Values followed by different letters in each column are different (*p* < 0.05) significantly.

**Table 2:** Particle size distribution of processed gari compared to four (04) commercial gari as reference collected in the local markets, *Abattoir Maroua* (Far North Region) and *Bertoua* (East Region) (Cameroon).

Harvest times	Gari	Particle size distribution (micrometer, *μ*m)									
		10	20	40	60	100	150	250	300	500	700
12MAP	TMS92	100.00	100.00	100.00	100.00	100.00	100.00	97.88	93.39	73.02	59.36
	TMS96	100.00	100.00	100.00	100.00	99.48	97.34	87.68	81.88	36.25	7.54
	IRAD	100.00	100.00	100.00	100.00	100.00	99.43	94.37	88.29	61.65	37.89
	EN	99.44	98.03	98.03	98.03	98.03	98.01	93.79	88.97	77.00	58.17
	AD	100.00	100.00	100.00	100.00	100.00	100.00	98.28	93.23	74.93	65.28

15MAP	TMS92	100.00	100.00	100.00	100.00	100.00	99.86	96.57	90.69	71.44	57.69
	TMS96	100.00	100.00	100.00	100.00	100.00	99.64	96.43	91.03	65.14	46.27
	IRAD	100.00	100.00	100.00	100.00	100.00	99.88	97.61	93.49	71.52	54.73
	EN	99.39	99.33	99.33	99.33	99.33	98.94	95.87	91.68	65.68	48.77
	AD	100.00	100.00	100.00	100.00	99.37	97.72	93.03	89.15	45.53	13.66

/	M1	99.48	90.97	79.66	63.41	52.62	52.33	42.69	30.76	14.41	5.75
	M2	100.00	100.00	100.00	100.00	100.00	99.85	97.24	93.80	78.06	57.59
	M3	100.00	100.00	100.00	100.00	100.00	99.73	97.07	93.08	79.36	61.33
	M4	100.00	100.00	100.00	100.00	100.00	100.00	99.60	97.36	84.35	77.51

12MAP: 12 months after planting (dry season); 15MAP: 15 months after planting (raining season); /: unknown months of the harvest. M1, M2, M3, and M4 were commercial gari collected from *Abattoir Maroua* and *Bertoua markets*, respectively.

**Table 3:** Identified FTIR peak values and functional groups of methanolic extracts of all gari samples.

Peak values	Functional groups	Peak values	Functional groups
3318-3436	Phenol	1648-1745	Aliphatic
3300-3696	Amine	1648	Amide
3001-3050	Aromatic	974	Alkene
3317	Amide	1415-1450	Aromatic
2832-2943	Aliphatic	1231-1283	Ether
2527-2693	Acid	1115	Secondary alcohol
2171-223	Amino acid	1020-1033	Sulfur

**Table 4:** Polyphenolic compounds (free and bound) of methanolic extracts of processed gari compared to four (04) commercial gari as reference collected in the local markets, *Abattoir Maroua* (Far North Region) and *Bertoua* (East Region) (Cameroon).

Harvest times	Gari	Free flavonoids (mgQE/100 gdw)	Bound flavonoids (mgQE/100 gdw)	Free tannins (mgTAE/100 gdw)	Bound tannins (mgTAE/100 gdw)
12MAP	TMS92	8.85 ± 0.49^d^	5.48 ± 0.12^d^	2.48 ± 0.03^e^	1.19 ± 0.07^cd^
	TMS96	8.37 ± 0.68^f^	8.64 ± 0.02^a^	4.28 ± 0.46^b^	0.89 ± 0.01^e^
	4114	10.16 ± 1.44^ab^	3.82 ± 0.48^g^	3.22 ± 0.14^cd^	1.38 ± 0.01^b^
	EN	8.23 ± 0.52^f^	2.42 ± 0.22^j^	2.41 ± 0.15^e^	0.92 ± 0.02^de^
	AD	6.62 ± 0.40^g^	7.89 ± 0.79^c^	3.57 ± 0.12^c^	0.67 ± 0.02^f^

15MAP	TMS92	8.59 ± 0.64^e^	8.85 ± 1.87^a^	4.42 ± 0.21^ab^	0.69 ± 0.08^f^
	TMS96	6.38 ± 0.05^h^	2.64 ± 0.58^i^	2.19 ± 0.04^f^	0.67 ± 0.02^f^
	4114	9.70 ± 0.63^c^	2.40 ± 0.26^j^	3.23 ± 0.02^c^	0.49 ± 0.01^g^
	EN	6.00 ± 0.58^i^	8.69 ± 1.57^b^	4.59 ± 0.12^a^	1.09 ± 0.03^d^
	AD	3.93 ± 0.41^l^	4.01 ± 1.74^g^	1.60 ± 0.04^g^	1.25 ± 0.02^ab^

/	M1	10.50 ± 0.14^a^	3.25 ± 0.59^h^	0.46 ± 0.00^i^	0.21 ± 0.01^h^
	M2	5.12 ± 0.35^j^	5.07 ± 0.08^e^	1.19 ± 0.05^h^	1.55 ± 0.01^a^
	M3	4.63 ± 0.28^k^	4.44 ± 0.06^f^	0.40 ± 0.17^i^	0.68 ± 0.03^f^
	M4	10.22 ± 2.50^a^	2.76 ± 0.01^i^	0.68 ± 0.01^i^	0.27 ± 0.01^h^

12MAP: 12 months after planting (dry season); 15MAP: 15 months after planting (raining season); QE: quercetin equivalent; TAE: tannic acid equivalent; dw: dry weight; /: unknown months of the harvests. M1, M2, M3, and M4 were commercial gari collected from *Abattoir Maroua* and *Bertoua* markets, respectively. Values followed by different letters in each column are different (*p* < 0.05) significantly.

**Table 5:** Antioxidant properties of methanolic extracts (free and bound polyphenolics) of processed gari compared to four (04) commercial gari as reference collected in the local markets, *Abattoir Maroua* (Far North Region) and *Bertoua* (East Region) (Cameroon).

Harvest times	Gari	FRAP (mgTE/gdw)		DPPH (*μ*gTE/gdw)		ABTS (*μ*gTE/gdw)		HRSA (*μ*gTE/gdw)	
		Free	Bound	Free	Bound	Free	Bound	Free	Bound
12MAP	TMS92	5.95 ± 0.67^c^	3.63 ± 0.21^cd^	11.69 ± 0.12^d^	7.42 ± 0.29^g^	26.66 ± 0.34^ab^	23.21 ± 0.61^de^	ND	17.95 ± 0.17^e^
	TMS96	4.89 ± 0.23^f^	4.32 ± 0.31^b^	6.17 ± 0.30^f^	4.15 ± 0.19^k^	24.72 ± 0.02^b^	31.93 ± 0.06^b^	ND	24.39 ± 0.59^c^
	4115	6.53 ± 0.12^b^	2.45 ± 0.06^h^	5.21 ± 0.23^h^	11.86 ± 0.30^d^	25.01 ± 0.12^b^	31.94 ± 0.12^ab^	2.58 ± 0.52^h^	20.09 ± 0.32^d^
	EN	7.97 ± 0.17^a^	2.27 ± 0.19^i^	11.91 ± 0.63^c^	8.16 ± 0.10^f^	20.43 ± 0.18^c^	25.00 ± 0.11^e^	10.47 ± 0.37^d^	21.79 ± 0.10^d^
	AD	5.37 ± 0.21^d^	1.78 ± 0.03^j^	13.05 ± 0.16^a^	4.76 ± 0.01^j^	21.86 ± 0.24^c^	29.60 ± 0.05^c^	2.83 ± 0.24^h^	18.17 ± 0.21^e^

15MAP	TMS92	4.71 ± 0.36^f^	4.59 ± 0.23^a^	12.01 ± 0.77^b^	6.08 ± 0.13^i^	29.71 ± 0.16^a^	34.39 ± 0.06^a^	7.12 ± 0.23^e^	25.50 ± 0.15^c^
	TMS96	3.78 ± 0.08^i^	3.36 ± 0.26^e^	4.94 ± 0.31^i^	10.09 ± 1.45^e^	28.18 ± 0.07^a^	25.67 ± 0.53^d^	13.91 ± 0.06^c^	35.09 ± 0.05^a^
	4115	3.83 ± 0.24^i^	2.78 ± 0.24^g^	10.09 ± 0.49^e^	6.35 ± 0.28^h^	28.52 ± 0.13^a^	29.80 ± 0.13^bc^	4.5 ± 0.11^g^	22.33 ± 0.42^cd^
	EN	4.32 ± 0.14^h^	2.33 ± 0.07^hi^	3.76 ± 0.11^a^	19.63 ± 0.14^b^	24.26 ± 0.71^a^	28.97 ± 0.10^c^	13.43 ± 0.72^d^	21.83 ± 0.35^d^
	AD	2.78 ± 0.01^k^	0.78 ± 0.06^d^	4.63 ± 0.25^j^	13.09 ± 0.44^c^	30.17 ± 0.10^a^	34.37 ± 0.06^a^	ND	29.48 ± 0.15^b^

/	M1	4.39 ± 0.12^gh^	3.12 ± 0.19^f^	4.48 ± 0.20^k^	3.05 ± 0.51^l^	23.02 ± 0.71^b^	28.96 ± 0.29^c^	5.49 ± 0.20^f^	12.25 ± 1.27^b^
	M2	5.01 ± 0.31^e^	3.58 ± 0.03^d^	10.04 ± 0.34^e^	2.70 ± 0.14^m^	26.29 ± 0.57^ab^	20.70 ± 0.59^f^	14.29 ± 0.15^b^	2.28 ± 0.01^b^
	M3	3.07 ± 0.12^j^	2.95 ± 0.21^g^	5.47 ± 0.23^g^	4.87 ± 0.31^j^	21.48 ± 0.59^c^	22.19 ± 0.51^de^	7.39 ± 0.63^e^	0.18 ± 0.02^f^
	M4	4.67 ± 0.38^g^	3.87 ± 0.53^c^	12.37 ± 0.14^b^	22.01 ± 0.44^a^	20.60 ± 0.07^c^	29.10 ± 0.10^c^	14.80 ± 1.15^a^	17.89 ± 0.23^e^

12MAP: 12 months after planting (dry season); 15MAP: 15 months after planting (raining season); TE: Trolox equivalent; dw: dry weight; ND: not detected; /: unknown months of the harvests. M1, M2, M3, and M4 were commercial gari collected from *Abattoir Maroua* and *Bertoua* markets, respectively. Values followed by different letters in each column are different (*p* < 0.05) significantly.

**Table 6:** Correlation between antioxidant activity, flavonoid, and tannin content in samples of cassava gari.

	Flaf	Tanf	ABTSf	DPPHf	FRAPf	HSRAf	Flab	ABTSb	Tanb	DPPHb	FRAPb	HSRAb
Flaf	1	0.065	-0.091	0.311	0.27	0.15	-0.102	0.282	-0.179	-0.035	0.45	0.113
Tanf		1	0.213	0.04	-0.209	-0.146	0.644^∗^	0.565^∗^	-0.172	0.15	-0.123	0.538^∗^
ABTSf			1	-0.083	0.047	-0.404	0.051	0.374	-0.092	-0.113	0.141	0.49
DPPHf				1	0.135	0.171	-0.078	-0.134	-0.441	-0.096	0.536^∗^	-0.044
FRAPf					1	0.595^∗^	0.077	-0.32	-0.208	0.071	0.638^∗^	-0.226
HSRAf						1	-0.131	-0.452	-0.18	0.224	0.312	-0.247
Flab							1	0.361	0.325	-0.104	0.156	0.047
ABTSb								1	-0.065	0.179	-0.239	0.613^∗^
Tanb									1	-0.443	-0.126	-0.152
DPPHb										1	-0.218	0.274
FRAPb											1	-0.239
DPPHb												1

f: free form of polyphenol; b: bound form of polyphenol; Tan: tannin; Fla: flavonoid. ^∗^Significant at *p* < 0.05.

**Table 7:** Factor loadings.

Factor loadings	F1	F2	F3	F4	F5	F6	F7	F8	F9	F10	F11	F12
Flaf	0.229	0.530	0.139	-0.165	0.739	-0.234	-0.053	0.078	-0.059	-0.074	-0.005	0.035
Tanf	-0.448	0.701	-0.124	-0.037	-0.303	-0.211	-0.291	-0.118	-0.161	-0.172	-0.018	-0.019
ABTSf	-0.493	0.257	-0.037	0.179	0.007	0.781	0.075	-0.016	-0.201	0.035	-0.015	0.020
DPPHf	0.457	0.386	0.152	0.688	0.036	-0.251	0.138	-0.140	-0.109	0.108	0.139	-0.008
FRAPf	0.660	0.397	-0.140	-0.447	-0.014	0.336	0.085	0.163	0.019	-0.062	0.190	-0.021
HSRAf	0.774	0.126	0.231	-0.318	-0.246	0.002	-0.314	0.028	-0.121	0.227	-0.064	0.005
Flab	-0.212	0.467	-0.641	-0.163	-0.440	-0.240	0.181	-0.009	0.076	0.086	0.032	0.035
Tanb	-0.499	-0.290	-0.429	-0.394	0.442	-0.079	-0.019	-0.306	-0.096	0.132	0.069	-0.016
ABTSb	-0.719	0.471	0.168	-0.080	0.130	-0.166	0.208	0.338	-0.025	0.138	-0.072	-0.028
DPPHb	-0.054	0.041	0.739	-0.436	-0.236	-0.091	0.356	-0.251	-0.062	-0.047	-0.010	0.006
FRAPb	0.623	0.575	-0.266	0.053	0.184	0.214	0.135	-0.246	0.134	0.006	-0.177	-0.018
HSRAb	-0.619	0.453	0.461	0.014	0.040	0.182	-0.252	-0.135	0.263	0.081	0.087	0.004

## Data Availability

Data are available on request (LA).
